# The Essentials of Multiomics

**DOI:** 10.1093/oncolo/oyab048

**Published:** 2022-02-22

**Authors:** John L Marshall, Beth N Peshkin, Takayuki Yoshino, Jakob Vowinckel, Håvard E Danielsen, Gerry Melino, Ioannis Tsamardinos, Christian Haudenschild, David J Kerr, Carlos Sampaio, Sun Young Rha, Kevin T FitzGerald, Eric C Holland, David Gallagher, Jesus Garcia-Foncillas, Hartmut Juhl

**Affiliations:** 1 Lombardi Comprehensive Cancer Center, Georgetown University Medical Center, Washington, DC, USA; 2 Georgetown University, Lombardi Comprehensive Cancer Center, Washington, DC, USA; 3 National Cancer Center Hospital East, Chiba, Japan; 4 Biognosys AG, Schlieren, Switzerland; 5 Institute for Cancer Genetics and Informatics, Oslo University Hospital, Radiumhospitalet, Montebello, Oslo, Norway; 6 Department of Experimental Medicine, TOR, University of Rome Tor Vergata, Rome, Italy; 7 JADBio Gnosis DA, N. Plastira 100, Science and Technology Park of Crete and Institute of Applied and Computational Mathematics, Foundation for Research and Technology Hellas, Heraklion, GR, Greece; 8 Personalis, Inc., Menlo Park, CA, USA; 9 Nuffield Division of Clinical and Laboratory Sciences, Level 4, Academic Block, John Radcliffe Infirmary, Headington, Oxford, UK; 10 Clinica AMO, Rio Vermelho, Salvador-BA, Brasil; 11 Yonsei Cancer Center, Yonsei University College of Medicine, Seodaemun-Ku, Seoul, Korea; 12 Department of Medical Humanities in the School of Medicine, Creighton University, Omaha, NE, USA; 13 Division of Human Biology, Fred Hutchinson Cancer Research Center, Seattle, WA, USA; 14 St. James’s Hospital/Trinity College Dublin, St. Raphael’s House, Dublin, Ireland; 15 Cancer Institute, Fundacion Jimenez Diaz University Hospital, Autonomous University, Madrid, Spain; 16 Indivumed GmbH, Hamburg, Germany

**Keywords:** cancer, multiomics, genomics, transcriptomics, proteomics, digital pathology, machine learning, artificial intelligence

## Abstract

Within the last decade, the science of molecular testing has evolved from single gene and single protein analysis to broad molecular profiling as a standard of care, quickly transitioning from research to practice. Terms such as genomics, transcriptomics, proteomics, circulating omics, and artificial intelligence are now commonplace, and this rapid evolution has left us with a significant knowledge gap within the medical community. In this paper, we attempt to bridge that gap and prepare the physician in oncology for multiomics, a group of technologies that have gone from looming on the horizon to become a clinical reality. The era of multiomics is here, and we must prepare ourselves for this exciting new age of cancer medicine.

## Teaser

Through multiomics, the combined use many available technologies, a more complete and dynamic vision of cancer can be obtained. This article bridges the gap between multiomics technology and oncology practice.

**Implications for Practice**

Tumors are constantly evolving, but so is our technology. Through multiomics—combined genomics, transcriptomics, proteomics, digital pathology, and other technologies yet to fully unfold—we can now obtain a complete dynamic vision of cancer. The resulting volume of data for any single patient is becoming so enormous that only artificial intelligence can decipher their clinical significance for use in the clinic. The potential impact on efficiency and patient outcomes is huge, but the practicing physician risks being left behind. In this paper, we attempt to bridge the gap between multiomics technology and oncology practice.

## Introduction

Cancer is a group of distinct genetic diseases that result from changes in the genome of cells in the body, promoting uncontrollable growth, metastasis, and disruption of normal physiological functions. This recognition, along with advances in technology, has led to the field of precision medicine, where the measurement of specific abnormalities in a specific cancer is galvanizing therapeutic strategies targeted to an individual instead of a population. As a result of continuous advances in molecular testing, including DNA sequencing techniques, the rapid expansion of actionable molecular targets, and a reduction of associated costs, molecular testing has become an essential element in the treatment decision-making of almost every cancer today. As more genetic testing is being done and many services now provide whole exome or even whole-genome sequencing, we recognize that the next phase of discovery will come from the measurement of post-translational molecular characteristics, including RNA, micro-RNA, protein, and phospho-protein levels. Technology has kept pace to enable the testing with a reasonable turnaround time. However, as we expand to broader testing, it is increasingly critical that the preanalytic quality of the tissues being tested is controlled; we recognize that tumor content and ischemia times can dramatically alter our results. Our clinical infrastructure will require changes to support this level of tissue management.

Within the last decade, the science of molecular testing has evolved from single gene and single protein analysis to broad molecular profiling as a standard of care, quickly transitioning from research to practice. Commercial services have rapidly expanded to provide practicing clinicians with the most up-to-date testing and researchers with fascinating data sets to explore. However, as the science expands and becomes more complex, the clinical reporting can fall out of date quickly. With this rapid transition has come a significant knowledge gap within the medical community. As more testing is done, it becomes increasingly important to understand the technologies themselves so that appropriate interpretation can be made at the bedside. Not only is the technology itself becoming more complex but also we now see a time where we are moving beyond genetics to proteomics and metabolomics. The amount of data obtained on any single patient is so large that we will become increasingly dependent on artificial intelligence to interpret the clinical significance of the findings. We predict that in the near future, tissues will be tested using multiple technologies simultaneously, termed “multiomics” , and we anticipate this will result in improved efficiency and outcomes in our treatment of cancer and other serious illnesses.

Having grown beyond the infancy of precision medicine but not yet into a fully matured science, we find ourselves in the awkward adolescents of scientific progress. As our ability to measure particular extensive molecular characteristics of an individual cancer grows, novel technologies including digital pathology and artificial intelligence emerge to support our ultimate decision-making process. Technologies that can measure circulating factors that predict resistance or sensitivity are being offered, in many instances without the clinician fully understanding the sensitivity and specificity of the test they are ordering. Never before in our medical history has technology moved so quickly. We in clinical practice are receiving complex molecular reports, which often leave us uncertain as to their clinical impact. In this paper, we provide an overview of the key elements critical to multiomic analysis so that the practicing clinician will better understand the implications on clinical practice today. While we predict that our future will ultimately become simplified and more precise, we must navigate the adolescent phase we are in to provide our patients with the best possible care.

## DNA: Next-Generation to Whole Exome to Whole-Genome Sequencing

### Technology

DNA is fundamental to life and to molecular profiling. Analytically speaking, DNA is more stable than RNA and protein, making it easier to measure and the logical place for the field of precision medicine to start. In oncology, our interest lies in cellular aberrations, and single gene mutation testing has had a major therapeutic impact on the treatment of patients living with cancer.^1^

**Figure 1.**
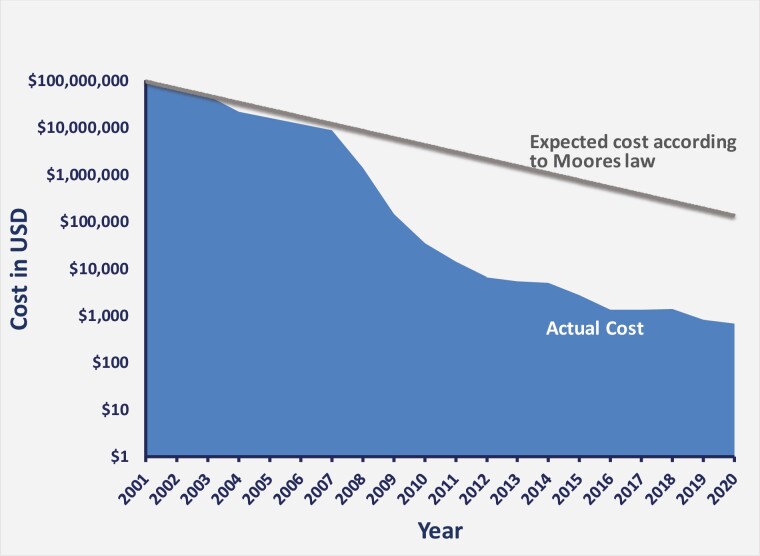
The decreasing cost of human genome sequencing over the last 20 years. The beginning of this millennium has seen the cost (in US Dollars) of sequencing per human genome decrease at a much greater rate than that predicted by Moore’s law—shown on a logarithmic scale using data generated by the National Human Genome Research Institute.^6^ The decline in cost began outpacing that expected from Moore’s Law at the beginning of 2008 when sequencing technology transitioned to next-generation sequencing (NGS) from Sanger sequencing.

The first sequencing techniques could sequence only short molecules of 20-30 nucleotides. The advent of next-generation sequencing (NGS) allowed expansion to whole-exome sequencing (WES), the sequencing of all exons (the protein-coding regions) in a genome, and whole-genome sequencing (WGS), which delineates the order of the total nucleotides in an individual’s DNA, thereby defining variations in any genome segment.^2,3^ Now it is possible to sequence hundreds of genomes a day in a single laboratory. NGS methodology is multipronged, including DNA fragmentation, library preparation, massive parallel sequencing, bioinformatics analysis, and variant/mutation annotation and interpretation (see *Next-generation Sequencing* later in this section).^4^ The technology has exploded due to advanced analytics and computing power, and the costs have fallen dramatically. Genome sequencing is beating Moore’s law many times over, and falling costs are the main driver in the journey toward wider adoption of these tools globally (Fig. 1).^5-9^ Today it is possible to decipher a genome (germline) for less than $500.

Advancing from single capillary reads to flow cells with billions of independently addressable DNA amplicons, multiple technologies compete on this multi-billion-dollar market, with a range of advantages and disadvantages. DNA and RNA can be decoded using short stretches of information for point mutations or gene expression (counting of molecules) or longer stretches for copy number and structural variation, including fusions or translocations. Of course, there is a whole ecosystem of tools and kits developed to facilitate the creation of these DNA and RNA “libraries of molecules,” and these are continuously evolving. Major government initiatives (ClinVar, ClinGen, COSMIC, and others) and overall global sharing of discovered disease-associated variants or other changes are also helping to create the framework necessary to interpret these results.

### Tissue Is the Issue

We often overlook that the backbone of high-quality, informative omics data—optimal tissue collection and fixation—is in the hands of healthcare facilities. The goal of fixation is to preserve cells and tissue components in as close to an in vivo state as possible, allowing confidence in diagnostic, prognostic, and predictive results. The importance of tissue handling is discussed later in this text (see *The Importance of Tissue Quality* section). Formalin-fixed paraffin-embedding is routinely used for clinical samples—it uses stable reagents and is relatively easy and accessible. However, although formalin-fixation cross-links macromolecules and stabilizes the tissue structure sufficiently for subsequent histological analysis, it allows degradation of nucleic acids and other small molecules.^10^ Freezing tissue in liquid nitrogen is optimal for molecular analyses because it literally freezes all molecular processes in time; however, ischemia time becomes increasingly critical to limit the degradation of molecules before freezing. Ideally, every healthcare facility should have a tissue collection and preservation framework in place, but this is not a standard that is established or maintained.

Most labs running genomic analyses have a pathological review protocol in place to assess tumor quantity and quality before proceeding. Often a biopsy sample contains a portion of tissues other than tumor tissue, and the labs will perform laser capture microdissection or macrodissection to improve the tumor cell yield.^11^ This extra step, when necessary, comes at a cost. The importance of the quality of the DNA extracted cannot be overemphasized as it affects the subsequent sequencing quality and final results.^12^

### Next-generation Sequencing

Commonly used NGS technologies can read only short fragments of DNA (less than 50-1000 bp), but newer technologies are slowly gaining traction and allow read length in the range of 10-100 kp. The obtained random short sequences are then mapped to a reference genome to create a consensus answer; the higher the number of reads accumulated for a particular sample and the deeper the depth of coverage, the more sensitive and accurate the assay will be.^13,14^

**Figure 2.**
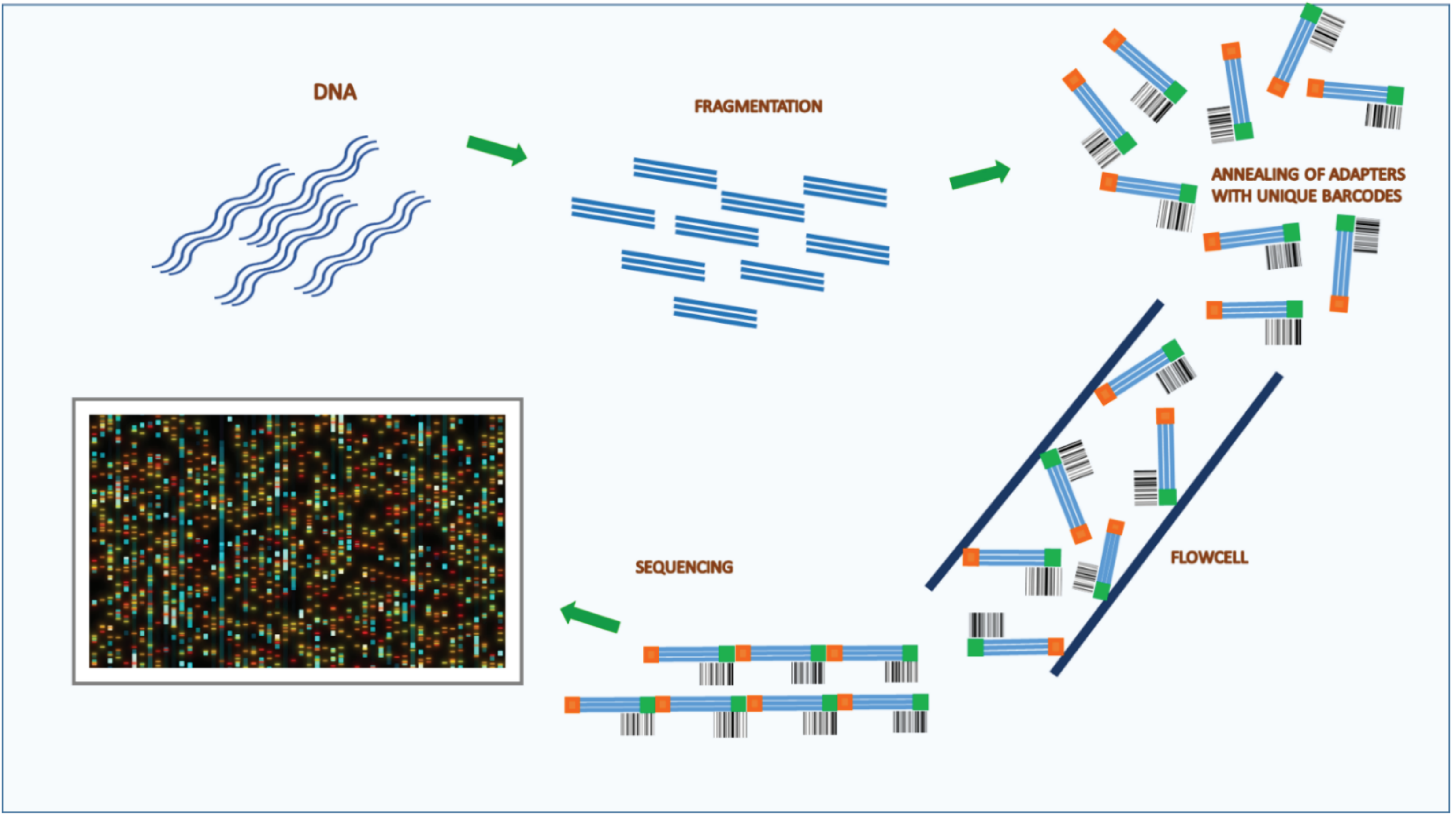
Next-generation sequencing (NGS). The first step of NGS is library preparation. A library is a collection of DNA fragments from the sample of interest with adapters (common short sequences with unique barcodes) annealed to their 5ʹ and 3ʹ ends. The adapters allow DNA fragments to attach to the sequencing flowcell, and their unique barcodes enable sample identification.

The first step of NGS is library preparation. A library is a collection of DNA fragments from the sample of interest with adapters (common short sequences with unique barcodes) annealed to their 5ʹ and 3ʹ ends. The adapters allow DNA fragments to attach to the sequencing flowcell, in turn enabling sample identification because of their unique barcode (Fig. 2).

To identify a tumor mutation, it is essential to have a high-quality library because poor-quality material will provide low yields. If the sample is of poor quality when sequenced, the diversity will drop significantly, and the depth of coverage will be substandard. Sometimes polymerase chain reaction (PCR) is used to amplify (generate millions of copies of) a particular region of DNA from an initially small sample. However, hybrid capture-based target enrichment is a superior method of achieving the same goal^15^; it uses probes (DNA or RNA single-stranded oligonucleotides) to enrich a particular region of the genome, which can then be sequenced deeply. The need for amplification and enrichment will be qualified in the report given to the oncologist. If the DNA is actually degraded, which frequently occurs with tissue fixation, the analyst will see a very high noise rate, which will mask rarer mutations.

### Impact

Physicians and patients are becoming more aware of the availability of genomic tests that might predict response to and affect the choice of therapy. However, there may be barriers. It is currently challenging for governments and insurance companies (depending on the region of practice) to adopt genomic testing universally because the value is still unclear; there is economic and humanitarian benefit in giving drugs that are going to work and avoiding those that are predicted not to work, but targeted therapies are expensive and genomic testing has thus far failed to clearly show worth in the majority of cases.^16^

### Challenges

The confusion lies in the value of single-gene testing versus small panel testing versus large panel testing (whole exome or whole-genome sequencing). How much is the standard of care, and how much is still experimental?

Clinical laboratories, governments, and insurance companies will probably have to work directly with pharmaceutical and biotechnology companies to lower genomic testing costs; testing could be made more affordable by cost-sharing.

There is a discrepancy between oncology practice and hereditary genetics practice regarding the identification of somatic versus germline alterations. There is a huge effort in hereditary genetics to interpret the functionality and clinical relevance of germline alterations. However, in oncology practice, tumor tissue, usually not accompanied by viable normal tissue, is sent out to the labs for testing. The clinician must then interpret the lab report and decide whether the findings are clinically relevant or not without knowing if certain mutations are somatic or germline.

As our knowledge grows, it is becoming clear that DNA sequencing cannot be our only predictive model. A recent example involves immune checkpoint pathway inhibition, which has seen many recent drug approvals. Microsatellite instability, tumor mutational burden (TMB), and PD-1/PD-L1 expression are often tested to guide these therapies. However, it has been found that unearthing variants or even the sum of variants may not always be enough to predict patient response to treatment.^17^ Cancers are found to modify an individual’s genome in a host of ways, including loss of human leukocyte antigen (HLA) alleles and major reprogramming of gene expression machinery.^18^ This means that more complex multiomic interrogations are necessary to make sense of any particular patient’s situation, which turns our current idea of molecular profiling on its head and makes payers very wary of the cost implications.

## RNA

### Technology

While DNA sequencing reflects the fundamental genetics of a tumor, RNA sequencing (RNA-seq; transcriptomic analysis) captures the current state of a cancer cell and is more reflective of the cancer’s biology. The transcriptome is the entire collection of RNA molecules or transcripts in a cell, critical to cellular function, including gene expression, coding, decoding, and regulation.^19,20^ Clinically speaking, messenger RNAs (mRNAs) and microRNAs (miRNAs) are currently of most interest as potential cancer biomarkers. mRNAs carry the sequences that code for protein synthesis. miRNAs are small non-coding regulatory molecules that are actively produced and serve as a regulatory control over other RNAs.^21^

The ability of RNA analyses to provide a dynamic, real-time measure of a patient’s cancer translates into predictive power beyond gene profiling alone. In their novel international WINTHER trial, Rodon et al used genomics combined with transcriptomics to match patients to treatments.^22^ After matching according to driver gene analysis, gene expression differences between tumors and normal tissues (mRNA; NGS) were assessed and used to tailor therapy based on a novel algorithm. The proportion of patients matched to treatment increased from 23% to 35% once transcriptomics was incorporated. The combination of DNA and RNA sequencing is now routinely used by profiling companies in biomarker assessment to predict patient cancer therapy.

Gene fusions and mRNA variant expression are among the molecular aberrations best defined using transcriptomics.^23,24^ Gene fusions (BCR-ABL being the most well-known) can result in altered production of RNA and the subsequent proteins and are more easily detected using RNA profiling, whereas they can be a challenge to detect when looking at DNA alone.^24^ One caveat is that RNA is more unstable than DNA, and hence its levels are more influenced by tissue work-up and storage methods; it cannot be emphasized enough that tissue quality and handling matter (see The Importance of Tissue Quality section).

As touched upon in the *DNA* section, the assessment of gene expression signatures (GES) using RNA-seq is calling our established treatment biomarkers into question. The retrospective analysis of tumor molecular data from the phase III JAVELIN Renal 101 trial^25^ of frontline avelumab plus axitinib compared to sunitinib in patients with advanced renal cell carcinoma challenged the standard biomarkers used to predict therapy.^18^ Hence, neither tumor mutational burden nor programmed cell death ligand 1 (PD-L1) levels but instead newly identified immunomodulatory and angiogenesis GES appeared to define progression-free survival.

Single-cell sequencing (scRNA-seq) is now possible, although still experimental and beyond the scope of this paper. scRNA-seq technology is being used to define intratumoral transcriptomic heterogeneity, which is important for therapeutic response, including the development of drug resistance.^26,27^

miRNA analysis is also still experimental, and expression levels are often assayed according to a particular research laboratory’s area of expertise. Methods include microarray analysis, real-time PCR (more quantitative than traditional reverse transcriptase [RT]-PCR), Northern blot, and in situ hybridization.^28^ Changes in miRNA expression have been shown to predict response to cancer therapy. For example, miRNA-126 levels in patient blood, which were observed to change during chemotherapy plus bevacizumab treatment, were proposed as a possible biomarker for resistance to anti-angiogenic-containing therapy.^29^ Additionally, circulating-miRNAs in patients with HER2-positive breast cancer could discriminate between patients with and without a complete response to lapatinib- and/or trastuzumab-based therapy, suggesting their use as an early non-invasive biomarker of response.^30^

The analysis of exosomal RNA in liquid biopsies and its potential uses in the oncology clinic are also under investigation (see Circulating Omics: Liquid Biopsies section).

### Impact

Gene fusions such as NTRK (site agnostic), BCR-ABL (chronic myeloid leukemia), or ELM4-ALK fusions (lung cancer) are best detected using RNA analysis. Historically, gene fusions have been assayed by fluorescence in situ hybridization and RT-PCR, but more recently, these highly clinically significant molecular predictors are being detected using RNA-based and DNA-based NGS assays.^31^ In a recent paper, ESMO guidelines were updated to recommend NTRK fusion assaying for “any malignancy at an advanced stage” but stated that the adoption of NGS-based methods in contexts other than academic laboratories was proving challenging, primarily due to costs, a relatively long turnaround time of 1-3 weeks, and the need for excellent tissue quality.^32^ However, several clinical labs have now adopted this technique as the standard.

Determination of GES using RNA-seq opens up new predictive biomarker avenues, and miRNA analysis has potential in predicting drug response and resistance, although it still has a way to go before becoming a clinical standard of care.

### Challenges

Tissue–based RNA is unstable, necessitating appropriate tissue collection and handling (see The Importance of Tissue Quality section). Interpreting RNA results and translating them into specific clinical recommendations will be a critical next step.

## Protein: The Proteome

### Technology

**Figure 3.**
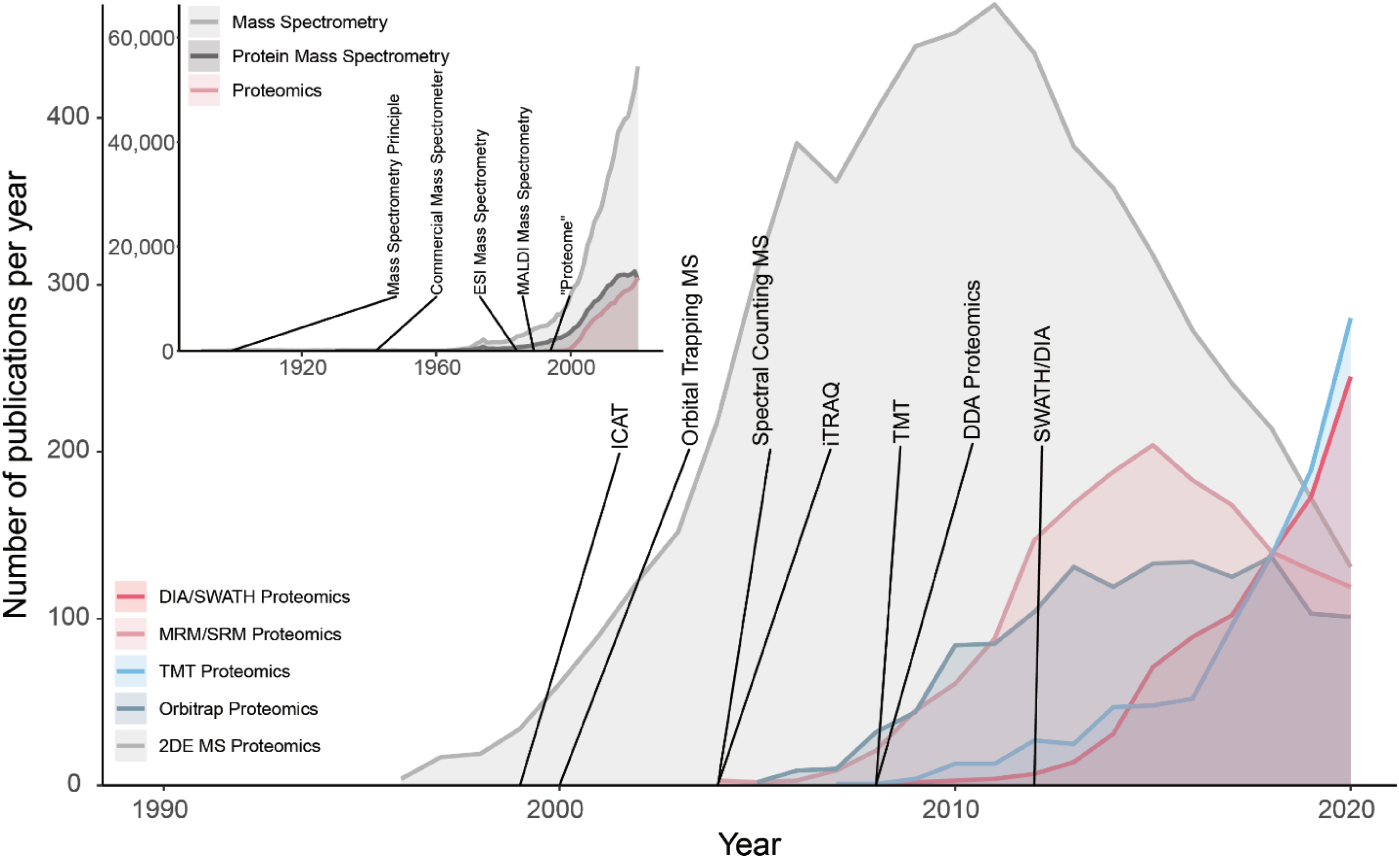
The recent success of proteomics approaches is linked to the availability of next-generation mass spectrometers. The number of publications per year for different proteomics-related approaches was obtained from PubMed. (Inset) The term “proteome” was coined nearly a decade after the invention of the mass spectrometry (MS) principle. Since the advent of electron-spray ionization and matrix-assisted laser desorption ionization commercial mass spectrometers, the use of MS continues to increase, including the application of MS for protein investigation and, more specifically, proteomics. Main: Early adoption of MS-based proteomic techniques was dominated by 2D electrophoresis (gray), followed by targeted analysis using multiple/single reaction monitoring (MRM/SRM, pink). Since 2010, modern and comprehensive proteomic techniques such as tandem mass tag (blue) and data-independent acquisition (DIA/SWATH, red) have revolutionized the proteomics field.

Proteomics—the study of all expressed proteins in a cell or tissue—is increasingly possible due to improvements in instrumentation and computer algorithms (Fig. 3) and is becoming increasingly available as a routine oncology technique. While laboratory tests based on single proteins, like HER2, PD-L1, and MSI, are already in routine use today, the shift toward measuring and interpreting entire proteomes is still in the early stages. Historically, proteomics has evolved from western blots and 2D electrophoretic techniques, which suffer from low throughput, limited quantitative precision, and lack of robustness. The quest for more in-depth analysis of cellular systems and increased quantitative ability, precision, and throughput led to liquid chromatography (LC) coupled mass spectrometry (MS) and only over the last 3-5 years, data–independent acquisition (DIA/SWATH) MS. Using mass spectrometers, antibody quality and specificity issues are avoided, and the protein(s) in any sample can be identified with great confidence due to an ability to cross-reference them with the actual molecular entities in a spectral library.

Traditional targeted MS–based proteomics methods (single and parallel reaction monitoring [SRM/PRM]) enable sensitive and consistent measurement of a low (1-100) number of proteins over many hundreds to thousands of samples. Data-dependent acquisition (DDA) methods allow deep proteome profiling up to more than 10 000 proteins per sample but lack reproducibility in cohorts of more than 100 samples. Modern DIA/SWATH methods combine the scalability and throughput of targeted methods with proteome depth of up to 10 000 proteins quantified with high precision in every run and are well suited for proteome-wide profiling of large cohorts of patients with cancer.^33^

Detection and quantification of post–translational modifications, such as phosphorylation, is also possible due to the high specificity of the MS spectrum. Importantly, profiling of phospho-proteomes requires the same instrumentation and workflow as used for global proteomics, although low abundance phospho-peptides require an enrichment step before the LC-MS measurement.^34^

### Importance of Normal versus Tumor Comparisons

The differentiation between tumor-specific proteome signatures and those that arise from inter-individual differences is vital in precision oncology. These “individual” signatures are especially pronounced in the plasma proteome.^35^

Several thousand proteins can be detected in plasma. Most of these are known, of high abundance (such as albumin and immunoglobulins), and usually not the proteins of interest; hence they can be depleted using an antibody column. After that, low abundance proteins can be analyzed. Plasma from patients living with cancer usually contains many more proteins than healthy plasma, and this in itself is interesting.

For tissue samples, the best way to control for inter–individual variability is the measurement of matched tumor and adjacent healthy tissue samples, in combination with tight control of sample quality. This matters for all kinds of analytes, such as DNA, RNA, and proteins. However, it is crucial to assure that tissue is truly “normal” for quantitative comparisons, such as protein expression. Studies have shown that adjacent “normal” tissue in close (less than 1 cm) proximity to the cancer tissue frequently has decreased expression levels compared to more distant tissue.^36^

Another critical point is that proteomic signatures in a sample usually only reflect the true biological state when the time between tumor excision and freezing in liquid nitrogen is less than 10 minutes. A prolonged ischemia time affects transcriptome and protein expression and phosphoprotein levels to a significant degree.^37-40^ Under these conditions, the normal tissue acts as an internal control for any particular patient, which should circumvent the issue of diurnal and any other kind of variation. There are strategies for obtaining normal tissue with tumor tissue, including sample microdissection and collection of normal tissue from a site distant from the tumor tissue during routine surgery. These collections are not part of standard practice and require a change in SOPs if proteomics becomes routine. Furthermore, it is not always clear which tissue serves as the “normal” comparator. For example, when testing a melanoma, normal skin is not the correct comparator tissue, and normal melanocytes would be impossible to isolate.

With further development in instrumentation and advances in computational interpretation, proteomics will likely become an important part of cancer treatment, supplementing the information obtained from genomics and transcriptomics.

### Impact

Cancer proteomics is expected to become a rich source of new targets and activated pathways for new therapies. We foresee a future where molecular reports include a kinome map of active pathways, driving the cancer biology, which could be used to predict drug response or cancer behavior. Deep data analyses will classify groups and subgroups, and the more data points available, the more accurate the predictions will be.

### Challenges

Quantitative proteomics has come a long way, but high sensitivity and throughput remain challenges. Additionally, we must create large and comprehensive databases to reflect and inform the oncology clinic.^41,42^

## Circulating Omics: Liquid Biopsies

### Technology

Blood samples contain circulating tumor cells, circulating tumor DNA, circulating tumor RNA, and exosomes. Single-cell testing is emerging as technically possible but still experimental and beyond the scope of this paper. Exosomes are tiny vesicles secreted by all healthy and abnormal cells and are abundant in body fluids^.43^ They contain protein, DNA, and RNA and are implicated in cell-to-cell communication and signaling. In this role, exosomes have potential as cancer biomarkers, analyzed in blood. Tumor-derived exosomes and their molecular cargo are under investigation as cancer prognostic markers, therapeutic targets, drug carriers, and maybe more. However, exosome analysis is still investigational and will not be detailed here.^44^

Currently, the most promising circulating biomarker for clinical practice is circulating tumor DNA (ctDNA). The technology involved in the analysis of ctDNA from patient blood has dramatically improved over the last few years in parallel with DNA analysis techniques in general—from single biomarker to multiple gene analysis in panels to NGS technology. The use of molecular barcodes enables multiple gene testing and comprehensive ctDNA analysis (Fig. 2).

#### Impact

In a study published in Nature Medicine in 2020, successful trial enrollment using plasma-based ctDNA sequencing (GOZILA project) was compared to that using tumor tissue sequencing (GI-SCREEN project).^45^ The patient accrual success rate was approximately 90% for both methods, but plasma testing has a shorter turnaround time than solid tissue testing—approximately 1½ weeks versus 1 month—and appears highly accurate. Archival tissue samples provide a snapshot of the molecular profile at the point in time of tissue harvesting, whereas liquid biopsies offer real-time information. Moreover, a single-site tumor biopsy is a relatively poor representation of the intra and inter–tumoral heterogeneity spectrum, whereas liquid biopsies can reflect this heterogeneity.^46^ As tumors progress and metastasize or are exposed to stressors (such as targeted inhibitors or chemotherapy), they develop acquired gene alterations that are not uniformly distributed throughout single tumors or within multiple tumors in the same patient.^46^ ctDNA is invaluable in detecting heterogeneous resistance alterations and can swiftly assist with subsequent therapy selection.

New technologies have been adapted to use blood samples to detect the presence or absence of cancer. Currently, clinical testing is available to detect minimal residual disease (MRD). However, this technology has evolved to allow cancer screening blood tests and real-time blood sampling to monitor for therapeutic benefit and development of treatment resistance. “Liquid biopsies” are simple to obtain and have the potential to transform much of what we do in cancer medicine today.

### Limitations

The primary challenge facing ctDNA analysis is tumor shedding—or the lack of it. Liquid biopsies do not provide any useful information in the case of a low or non-shedding tumor.^47^ Even in higher shedding tumors, fusion drivers cannot be detected using liquid biopsy due to low sensitivity. Furthermore, genotyping is not possible with ctDNA, and aging poses a problem because it is strongly associated with clonal hematopoiesis (CHIP), which leads to normal cell mutations that can easily be misclassified as tumor-derived mutations in genes such as KRAS, JAK, and several others during ctDNA analysis.

Exosome analysis may overcome the problem of low-shedding tumors, and research is underway to combat other challenges.

## Artificial Intelligence

### Technology

Artificial intelligence (AI) has offered medical expert systems, ontologies, and numerous other technologies to fight cancer. However, the sub-field of AI most relevant to cancer multiomics is machine learning, which encompasses deep learning (see the Digital Pathology section as an example). Machine learning’s purpose is to transform data and observations into knowledge and evidence-based medical decisions.

In applying machine learning to multiomics, the aim is to learn a predictive model that, given multiomics measurements, can predict an outcome of interest in new samples, patients, or tissues. The term “outcome” is used as a general term to denote the quantity we would like to predict. Typical outcomes are the type of disease, disease status, a subtype of cancer, time to an event of interest (death, metastasis, relapse, complication, diagnosis), and response to therapy. In addition, feature selection filters out irrelevant and redundant markers to identify the set of markers (called a signature) required for optimal prediction. Differential expression analysis considers the pairwise correlation (mutual information, predictive value) of a marker with the outcome, independently of any other marker. In contrast, typical machine learning and feature selection algorithms consider the correlation (predictive value) of a marker in the context of other markers. They consider the values of markers combinatorially when creating a predictive model. This is arguably the major conceptual difference between the 2 methodologies. Machine learning can sort through hundreds of thousands of markers to find a signature and a possibly non-linear predictive model. The model can be used to inform clinical decisions (eg, change therapy if it is predicted to be ineffective); the markers in the signature can be used to design diagnostic assays, point to plausible drug targets, and provide intuition to the underlying biological mechanisms involved.

AI is increasingly used now because, today, we have ample computing power, more data, richer data, and public data. Just a few years ago, multiomics datasets were practically nonexistent, while now they are relatively common. Moreover, AI as a field has made theoretical and algorithmic breakthroughs in the last couple of decades. Essential AI infrastructure has been built, which consists of medical and biological ontologies and knowledge bases and allows the incorporation of prior knowledge into the algorithms.^48,49^

### Impact

Multiomics using AI is just now beginning to impact clinical care, relying on 2 methodologies. The first one is automated machine learning (AutoML). AutoML platforms automate the machine learning process end-to-end, promising to democratize it to clinicians, drastically boost productivity, and reduce errors. An oncologist can perform a sophisticated and, more importantly, correct machine learning analysis of their data automatically, provided they use accessible data formats and suitable, validated tools.^50^ A second one is the development of methods to integratively analyze large portions of available data and make clinical sense of them. One oncologist may focus on breast cancer and another on mesothelioma, and, traditionally, each dataset is studied independently. Although there will be many omics profiles, all these data are obtained on the same system, namely humans. They involve the same genes that follow the same biological rules, constraints, and patterns. A dataset on one disease may contain useful information when analyzing another. For example, in recent work, researchers created maps of thousands of gene expression studies discovering biological similarities between different phenotypes and diseases at the molecular level.^51^ Future AI will allow us to process all the available data gathered on humans, find all the hidden patterns, and provide a global view of the inner workings of cells, tissues, and organs.

### Challenges

AI in multiomics is currently a precious and costly resource because human experts are scarce and expensive. Simply put, the data are produced at a faster pace than we can graduate experts. As a result, most data generated are not analyzed or fully tapped for their informational content. Other intangible costs are related to AI being complicated and error-prone. It often uses a black box, obscuring understanding and making AI solutions harder to verify and apply an expert’s domain knowledge. Hence, not only is it easier for analysts to make mistakes, but it is harder for clinicians to correct them. The phenomenon where a predictive model is published as having excellent predictive power but then fails to generalize to new samples, also known as overfitting, is all too common, even among papers published in the most prestigious journals.^52^ Do not trust and always verify.

AI researchers will keep spawning new and wonderful algorithms by the day, but these algorithms need not only data but semantically annotated data—beyond the simple annotation schemes now available in most common biorepositories. Interpretability and explainability of AI-derived results are critical issues and essential AI research directions. Security and privacy are prerequisites for any analysis of personal data.

Finally, we need the democratization of AI for oncologists. AI for multiomics requires significant expertise, time, effort, and cost. At present, only well-funded projects, teams, and companies can provide oncologists with sufficient expertise to be productive. Tools that allow non-experts to derive correct results are needed.

## Digital Pathology

### Technology

Digitalization of pathology is long overdue because the standard practice in pathology still uses a 100-year-old technology. In digital pathology, glass tissue slides that would once have been viewed only under a microscope are inserted into an image scanner and saved as an image file for digital viewing on a computer. Digital slides can be read remotely and stored in EMRs, and the images can be used to diagnose molecular results.^52^

**Figure 4.**
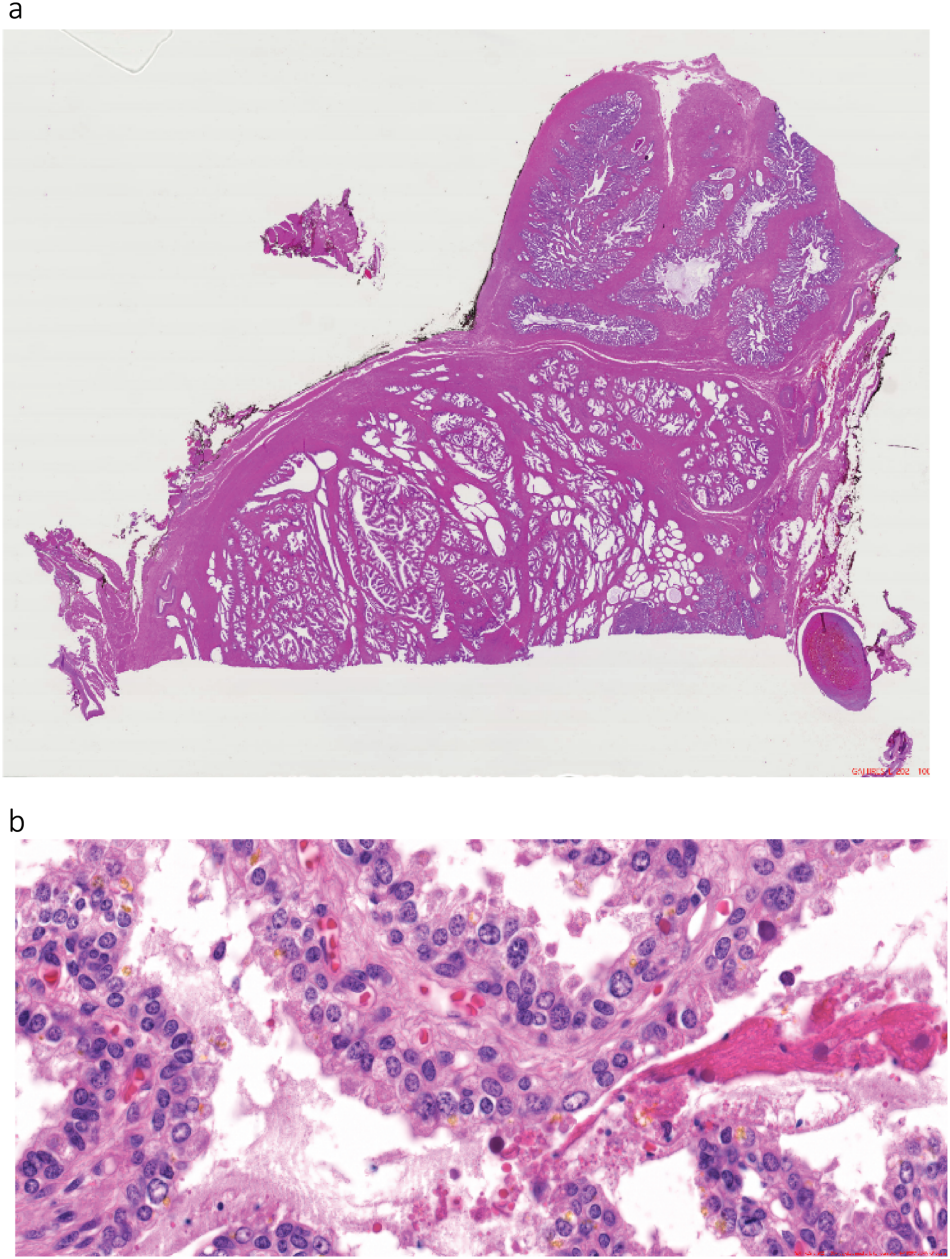
Digital images of prostate cancer. (**A**) Whole slide, prostate cancer. (**B**) High resolution of a section of the slide.

It is not pathology per se but the technology applied to pathology slide analysis that has evolved; digital pathology (Fig. 4) is being combined with AI to make it a much stronger discipline.^53^

## Digital Pathology and Deep Learning

As explained in the Artificial Intelligence section, AI has the subtopic of machine learning, and machine learning has the subtopic of deep learning. If, for example, a researcher has tumor tissue slide images from 200 cases with a good prognosis and 200 cases with a poor prognosis, a computer can find the differences in these images using deep learning. By interrogating pixels in digital pathology using sophisticated deep learning methods, we can actually train a machine to extract the necessary information from the images to distinguish between good and poor prognoses in a highly reproducible and objective way, reducing the interindividual variation in conventional pathology reporting. It may be considered that deep learning is the true AI because it selects the important features captured from the digital, pixelated landscape.

The quality of slide images for the deep-learning process—their resolution, or the number of pixels in a defined area—has been in existence for the last 20 years. Other analytical technologies had to evolve in parallel so that large data sources could be mined optimally. Most significantly, a great deal of computational power and an efficient way to handle big data are required for deep learning in digital pathology. This has now all fallen into place.

Digital pathology and deep learning will not make the pathologist obsolete; quite the opposite. Pathologists will become more contributory players in personalized medicine than is currently the case. This natural evolution, embracing objective and quantitative image analysis, permits more precise prognostic information to be made available to the multidisciplinary team.

### Impact

Based on the same routine tissue sections, deep learning can extract so many different features from an image that it is now possible to classify a patient by prognosis as well as diagnosis.

Although tumor molecular profiling has had some predictive success, its impact on most patients with colorectal cancer has been limited. The molecular biology of colorectal tumors was explored using DNA sequencing and RT-PCR in paraffin-embedded tissue. The genetic algorithms developed, which focused on known oncogenes and canonical signaling pathways, were relatively weak. Hazard ratios were somewhere between 1.5 and 2, and the algorithms were not quite enough to guide our treatment decision-making.^54^ We still were unsure whether we should or should not offer adjuvant therapy, whether we should or should not intensify treatment, whether we should or should not look at the duration of therapy we gave. While acknowledging the progress made in other tumor types, after 10 years of looking at the somatic tumoral mutation landscape and RNA transcriptomic profiling in colorectal cancer, the tool that ended up being sufficiently clinically useful in defining patient prognosis and enabling clinical decision-making in terms of hazard ratio and statistical power was essentially the 100-year-old discipline of pathology, albeit upgraded by digitization and deep learning. At this point, it should be noted that advancements in digital pathology and multiplex staining of tissues make the combined microscopic evaluation of various markers in parallel possible; hence the discipline can be quite powerful.^55^

A recent study illustrates the potential of deep learning coupled with digital pathology, wherein scanned images of H&E-stained primary colorectal tumor tissue from patients with stage II and III disease were coupled with computerized free learning to develop a powerful biomarker of outcome.^56^ To this end, several million image tiles from 4 cohorts of patients were processed. The patients were separated according to whether or not they had a distinct outcome—a distinct outcome could be either good or bad. Image tiles from patients with distinct outcomes facilitated deep learning. Data from the patients with indistinct outcomes then allowed the development of a prognostic biomarker, which was subsequently tested on over 900 patients and independently validated in another thousand or so patients treated with single-agent capecitabine. The biomarker had the potential to guide adjuvant treatment selection according to which patients were at low risk from their disease and could avoid therapy and which patients needed more aggressive treatment.^56^ This marker was more potently prognostic than any existing genomic or traditional marker.

All the other comparative methods in genomics typically mean another sample taken from the patient and added costs of laboratory processing, including reagents and probes, and considerable analytical work and time. Here, we take a digital image of an existing routine slide from the pathology department and analyze it; the analysis takes only one minute, can be done remotely on a laptop with a GPU card, has little to no additional costs, and necessitates no additional sampling from the patient. Genomics and traditional marker assessment still have a place in diagnosis and prediction of patients living with cancer; only they have much greater potential when combined with digital pathology in the field of multiomics.

## The Importance of Tissue Quality

### Technology

If we intend to personalize care for patients with cancer, we must also objectively care for their tissue samples. Molecular profiling, including assessment of biomarkers to adequately stratify a patient’s cluster and therefore guide cancer therapy, require that tissue samples accurately represent a patient’s tumor biology and not surgical manipulation or ischemia-induced alterations in gene expression and protein phosphorylation.^37^*Preanalytical variables lead to biomarker instability*, impacting comprehensive molecular analyses and patient tissue phenotyping.^57^

Updated standard operating procedures (SOPs) for tissue collection and handling must be in place to guide personnel in the swift excision and preservation of tissue for diagnostic purposes, in obeyance with histopathology rules, while also immediately freezing a portion of the sample for multiomics study.^37,57^ Essentially, speed and efficiency are required as unfrozen tissue outside of the body for more than 10 minutes might be compromised when considering molecular analyses.^37^ This is of particular relevance, for example, when analyzing gene expression and phosphoproteomic data to avoid spurious and misleading results. Implementing these updated tissue collection and preservation procedures as a standard practice requires that dedicated trained personnel are in the surgical suite, committed to tissue quality and procuring an optimal frozen tissue sample while interfering as little as possible with the routine workflow of the surgical suite.

This kind of tissue collection has not been the standard of care to date because, until recently, it has not been seen as a critical component of multiomics analyses. In fact, in this respect, most surgical suites still operate as they did in the nineteenth century because the current standard of care is built on morphological analysis of histological sections. Tissue morphology is less sensitive to change than is tumor biology—the cellular interaction of molecules. Also, it appears that the impact of surgical manipulation and ischemia on the molecular composition of tumor tissue is more significant than seen for normal tissue, probably because tumor cells are generally more active than normal cells.^37^

*The collection of normal tissue at the same time as tumor tissue is critical*. This matched tissue collection serves as a much-needed comparison methodologically, allowing time-sensitive variations to be separated from true tumor tissue anomalies. In addition, the corresponding normal tissue type should be well defined to obtain truly comparable tissue pairs. For example, colon cancer develops from epithelial cells of the mucosa. Therefore, only mucosal tissue represents the normal control. The entire colon wall contains muscle cells primarily and, thus, is not a meaningful normal control.

Radiology-guided tissue collection minimizes ischemia time but provides only small pieces of tissue, which dry out more quickly than a larger piece of tissue. Adequate collection and preservation speed are therefore imperative.

If the decision is made to fix tissue sections in formalin, it should be considered that tissue quality is not reliable for many newer molecular tests because it takes about an hour per 5 mm of tissue for the formalin to preserve every cell in that tissue sample. Freezing is a requirement of any test that is not focused on a few well-defined proteins of known stability. Moreover, if the aim is to integrate complex data points to get a much more defined picture of the tumor, as we can now do with bioinformatics and AI solutions, then only liquid nitrogen freezing of tissue is acceptable.

### Impact

As incorrect tissue handling can dramatically impact basic test results, it may lead to a completely different patient prognosis and therapeutic track. Most clinical hospitals are not set up for the kind of precise tissue collection described here. However, until these specially developed SOPs are in place (globally), the validity of diagnostic testing that goes beyond basic genomics should be questioned, and patient care and safety may be jeopardized.^37,57,58^

Using immunohistochemical analysis, Koury et al showed that estrogen and progesterone receptor immunoreactivity and HER2 positivity were lost after a delay of time-to-formalin fixation of 1-2 hours, leading to false-negative results.^59^ Considering time-to-formalin fixation was routinely 2-3 hours, the impact on patient therapy was dramatic. Other targets that serve as diagnostic biomarkers, such as EGFR, are also affected by surgical ischemia time, and using them to guide treatment is questionable under current tissue collection and fixation guidelines.^37^ The 2010 paper by Nkoy et al^60^ reports on their retrospective analysis of this concept across several hospitals, demonstrating that Friday and Saturday surgeries led to less accurate detection of estrogen and progesterone receptors in breast tumor tissue by immunohistochemistry—potentially negatively impacting patient treatment. The breast cancer community paid attention to these data, and the American Society of Clinical Oncology and the College of American Pathologists drew up preanalytical procedural guidelines, which can be found in the Accreditation Checklist of the CAP Laboratory Accreditation Program.^58^ However, these breast cancer guidelines stand alone and this wisdom is yet to find its way into other disease-group guidelines. In addition, an ischemia time window of 1 hour (as recommended by the CAP guidelines) still jeopardizes the analysis of many potential biomarkers and is rather a compromise between clinical practicability and scientific requirement for advanced analytics. The future of immunotherapy is dependent on the assessment of multiple biomarkers. When assessing numerous signatures using different technologies, preanalytical factors play even more of a significant role in ensuring accurate prediction of clinical outcomes. This concept is reflected in recent guidelines for biomarker validation in cancer immunotherapy.^61^

Considering biomarker expression levels vary because of handling errors or slow fixation time by formalin, the powerful technologies discussed in this review will remain limited and can only be applied to a few highly stable tissue biomarkers. Moreover, now that we are trying to understand numerous molecules in parallel, if we genuinely want to see progress in oncology, standardized rapid collection and liquid nitrogen-preservation of tissues inarguably form the only sturdy foundation on which to build.

### Challenges

Transporting this research-based effort to regular practice poses a challenge. Facilities for this kind of rigorous sample collection and work-up, including frozen treatment, are not universally available in surgical suites.

A workaround using PAXgene fixation and other fixative reagents is under investigation. PAXgene methodology works for blood samples, and the use of other novel solid tissue fixatives is affording significant improvement over traditional fixation methods. However, fixation methods still do not have the edge over tissue freezing in liquid nitrogen and potentially lead to a loss of molecular information. The incorporation of more stringent tissue collection and preservation methods into official guidelines is probably still in the distant future, but raising awareness today is vital. Specialized cancer centers are able to apply the required rigorous standard of tissue handling for multiomics analysis, and some have already become beacons for the development of multiomics data analysis for personalized medicine to its full potential. By demonstrating medical value for their patients, the official guidelines might change and allow reimbursement—the main barrier for implementing higher standards.

The techniques and instruments for multiomics analyses are available. The flow and the process now need to change. Until then, there will be a loss of multiomic information and progress.

## Ethical Considerations in Multiomics

One of the many lessons learned from the Human Genome Project was the importance of integrating ethical deliberation into emerging translational science.^62^ This process not only facilitates the development of health policies that are scientifically and ethically sound but also fosters discussion amongst various stakeholders about the desired goals of implementing new technologies. As with any new technology, including multiomics tests, many of the looming questions can be just as important as the ultimate answers.

For example, what is the goal of precision medicine? Is it better health, more knowledge, or the identification of optimal treatment options? Clinicians, researchers, and patients are likely to have different answers. Moreover, responses vary depending on societal, community, cultural, and individual perspectives. Thus, discussion amongst all stakeholders is critical.

Indeed, an informed consent *discussion* is central to eliciting the patient’s and the clinician’s goals and expectations. From a patient’s perspective, the purpose of pursuing testing with multiomics technologies may be simply “to get better” or “to have more time with loved ones.” From the clinician’s perspective, the goal may be to determine the treatments most likely to give the patient a good response. However, a good response does not necessarily translate to what the patient desires regarding a satisfactory quality of life or restoration to a prior state of “health.”

In addition, selecting a treatment course when there are multiple options can be challenging. Clinicians may aim to enroll patients in clinical trials, in which the overarching goal is to acquire data that will guide future treatments for specific types of patients. Thus, for an individual patient, there may be little or no added benefit from participating in research relative to standard care, but research patients are not getting less than standard care in most trials. This concept is often not well understood by patients; likewise, it is often difficult for clinicians to convey.^63^ In addition, some treatments may be inaccessible due to cost or other logistical considerations. It is also possible that multiomics analyses reveal no therapeutic targets, although standard therapies and research trials may still be appropriate options to consider.

Another consideration is that tumor genomic profiling (ie, somatic testing) to identify therapeutic targets is increasingly paired with germline testing. Patients need to understand that while pathogenic germline variants may impact treatment selection, such testing can reveal risks for other cancers and risks relevant to their relatives.^64^ Some variants may be consistent with the patient’s phenotype (eg, a *BRCA2* variant in a patient with pancreatic cancer), whereas others may be unexpected (eg, a *BRCA2* variant in a patient with lung cancer and no family history of cancer). Moreover, as testing expands to the whole-genome, pathogenic variants will be identifiable in many cancer and non-cancer-related genes.^65^ How much of this information will be disclosed to patients, and through what process? Do patients have the right to *not* know about the presence of specific genetic variants? Patients cannot opt *out* of test results unless they know and understand their significance.

Research is central to progress in Western medicine, which is why many patients offered multiomics testing as part of their clinical care are also asked to allow their biospecimens and data to be used in research. Research usually necessitates data sharing, sometimes with industry partners,^66^ and herein lies a problem because many patients have concerns about privacy and data sharing. Transparency and trust-building are crucial to promoting patient autonomy and engagement.^67^

Finally, current results will quickly become outdated. Is there a responsibility to re-analyze and reinterpret data and then recontact the patient or family members about newly identified germline pathogenic variants or other recently discovered clinically significant information?^68^ Such disclosures can cause anxiety and confusion, and it is important to clarify expectations before testing as part of the informed consent process.

To the extent that we can anticipate these and other ethical challenges, clinicians and researchers will be better equipped to empower patients to make decisions that will best address their healthcare needs and goals.

## Discussion

The standard of care in medicine is not the ceiling we reach for but the floor on which we stand. It represents what we have achieved to date. Often, changes in the standard of care come slowly, incrementally, like climbing stairs one floor at a time, with breaks in between to catch our breath. The pace at which precision medicine norms are changing is much more of a steep incline—rapid progress if you can keep your footing; unfortunate falls if not. In this overview of precision medicine 2021, we have emphasized the technologies we will increasingly rely on: the strengths and the weaknesses. As practicing cancer specialists, it is critical that we understand the technology as it will increasingly fall on us to interpret the results. Equally important is the development of clinical reports that are both all-encompassing and user-friendly. These may use visual depiction—imagery instead of text—but whatever format they take, treating physicians’ next steps in the treatment of their patients should be clear.

**Figure 5.**
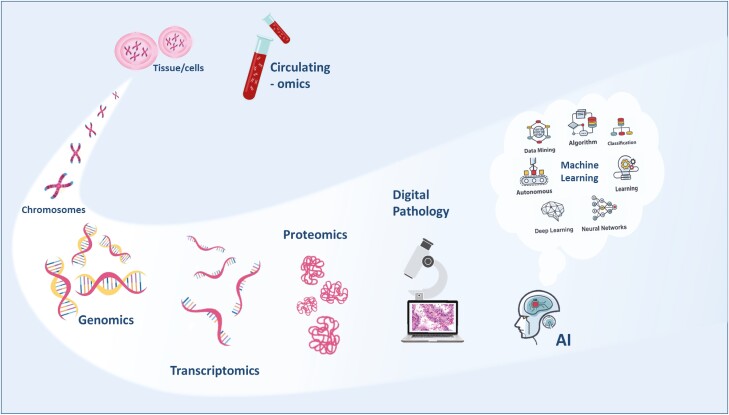
Cancer multiomics.

Our primary conclusions are that precision medicine will continue to expand to a multiomic profile, and the multiple omic layers will provide the oncologist with a complete dynamic vision of cancer, the capture of which relies wholly on preanalytical and assay quality. Artificial intelligence, machine learning will be the fundamental tool in processing and translating these molecular data, providing us with the prognostic and predictive answers we seek (Fig. 5). We also conclude that multiomic analyses will give value to our healthcare systems, lowering costs through defining more efficient treatment decision-making. As such, multiomic profiling will rapidly become the new standard of care for us all, and once we reach that point, maybe we will find a moment to look around, take in the view, and catch our breath.

## Data Availability

The data underlying this article will be shared on reasonable request to the corresponding author.
